# Parametric and kinetic study of solvent-free synthesis of solketal using ion exchange resin

**DOI:** 10.55730/1300-0527.3376

**Published:** 2022-02-23

**Authors:** Dheer A. RAMBHIA, Sravanthi VELUTURLA, Archna NARULA

**Affiliations:** Department of Chemical Engineering, M.S. Ramaiah Institute of Technology, Bangalore, India

**Keywords:** Glycerol, solketal, process parameter, ion exchange resin, kinetics

## Abstract

The ketalization reaction of glycerol with acetone for solketal formation was performed using a gel-type ion exchange resin, Indion 225H in a solvent-free medium. The experimental parameters molar ratio, catalyst loading, rate of stirring, and temperature were analyzed and evaluated for their impact on the conversion of glycerol. A kinetic model based on the Langmuir–Hinshelwood–Hougen–Watson (LHHW) equation described the kinetics of the heterogeneous system. The parameters were evaluated using the ode45 solver and Genetic Algorithm (GA) optimization function on MATLAB software. The activation energy for the ketalization of glycerol was found to be 39.3 kJ/mol, the enthalpy of adsorption of water was −23 kJ/mol. The present work provides an economical path for the valorization of biodiesel-derived glycerol.

## 1. Introduction

The transesterification of vegetable oils and methanol in the presence of an acid or base catalyst leads to the production of biodiesel and glycerol [1]. This transesterification reaction has a selectivity of 90% for biodiesel and 10% for the by-product glycerol [2]. The crude glycerol has hindered the commercial value due to the presence of inorganic salts and impurities [3,4] resulting in huge stacking of crude glycerol with an estimated of worth 41.9 billion liters of crude glycerol by 2020 [5,6]. A techno-economic study on glycerol purification reported that an exclusive purification of glycerol is not economical unless integrated with a process of producing specialty chemicals like fuel additives [7].

The valorization of glycerol to value-added products employs routes such as hydrogenolysis [8,9], Oxidation carbonation [10], etherification [11], esterification [12], acetalization/ketalization [13,14]. The ketalization of glycerol using acetone produces solketal (2, 2-dimethyl-1, 3-dioxolan-4-yl) as the main product and 2,2-dimethyl-1,3-dioxan-5-ol as the side product. Solketal can be used as a fuel additive as it reduces the particulate emissions [15], improves oxidation stability [16], enhances the octane number of fuel [17], and also improves the cold flow properties of liquid fuels [18]. Studies on the ketalization reaction were performed using heterogeneous catalysts like Amberlyst resins [19], zeolites [20], polymers [21,22], and organic–inorganic [23]. Amongst these gamuts of catalysts, ion exchange resins are gaining popularity as they show high activity, availability, reusability and a noncorrosive nature.

For any industrial application, it is significant to consider the use of commercially available and economical catalysts. The use of solventless condition is environmentally benign and economical for industrial applications due to lower separation costs.

In view of this, the present work highlights the use of a commercially available and economical gel-type ion exchange resin Indion 225H for the ketalization of glycerol to solketal in solventfree conditions. With this objective, optimal operating conditions were established and a kinetic model for the reaction was developed based on the Langmuir-Hinshelwood-Hougen-Watson (LHHW) equation. The experimentally obtained data was fitted to kinetic model using ode45 solver and Genetic Algorithm (GA) in MATLAB software. The present work provides an economical path for the valorization of biodiesel-derived glycerol.

## 2. Experimental section

### 2.1. Materials

Glycerol and acetone (90–95 wt% purity) were procured from Sd. Fine Chem. Solketal [(s−) (+) – 1,2-isopropylideneglycerol, 99wt%] was procured from Sigma Aldrich Pvt. Ltd. The cationic ion exchange resin Indion 225H was obtained from Ion Exchange (India) Pvt. Ltd. The characteristics of the catalyst are given in Table 1.

### 2.2. Reaction procedure

The glycerol ketalization was accomplished in a 100 mL three-neck round bottom flask placed inside an oil bath equipped with a magnetic stirrer and a hot plate. A condenser was attached to the round bottom flask to condense and reflux the vapors in order to maintain constant reaction volume and avoid loss of reactants. The temperature was regulated using a thermostat and thermocouple placed inside the flask. The reactants were heated and stirred vigorously until the desired reaction temperature and homogeneity was achieved. In a typical experimental run, 10.0 g of anhydrous glycerol, 12.5–25.0 g of acetone at variant mole ratios of (Glycerol: Acetone) G:A were fed into the reactor and precalculated amount (0.29 g) of catalyst was charged after attaining the desired temperature. The reproducibility of results was checked by repeating the experiments thrice and the average values were reported. Samples were collected every 15 min for a reaction time of 180 min and were analyzed by a GC-FID (Mayura Analytical LLP Model 1100). The reaction parametric studies were performed for the molar ratio of G:A of 1:2, 1:3, and 1:4, the temperature of the reaction was 298 K to 323 K, and catalyst loading ranged from 0.25 to 2.0 wt%.

### 2.3. Product analysis

The main components in the product were detected with a gas chromatograph equipped with FID (Mayura Analytical LLP Model 1100). The column used was the Carbo-Stabil wax column, using helium as the carrier gas. The injector temperature is maintained at 120 °C for 3 min then increased to 250 °C at a ramp rate of 50 °C/min. The detector temperature was maintained at 250 °C. The run-time per sample was 10 min. The peaks were compared with the pure solketal.


(1) $$\text{Yield }(\text{mole}\%)=\frac{moles\;of\;solketal\;formed}{Initial\;moles\;of\;glycerol}\times 100\%$$

(2) $$\text{Conversion }(\text{mole}\%)=\frac{initial\;moles\;of\;glycerol-final\;moles\;of\;glycerol}{Initial\;moles\;of\;glycerol}\times 100\%$$

(3) $$\text{Selectivity }(\text{mole}\%)=\frac{moles\;of\;solketal\;formed}{Total\;moles\;of\;product\;formed}\times 100\%$$

## 3. Results and discussion

The reactions were conducted at various stirring speeds to investigate the effect of mass transfer resistance on the reaction kinetics. The composition of G:A–1:3 at 323 K and 1.0 wt% catalyst loading were subjected to stirring speeds of 300, 700, and 1000 rpm. The tests gave the same conversion of 70% as well as a constant reaction rate at different time intervals. Hence the reactions were carried at 700 rpm.

### 3.1. Effect of feed composition on solketal synthesis

**Figure 1:**
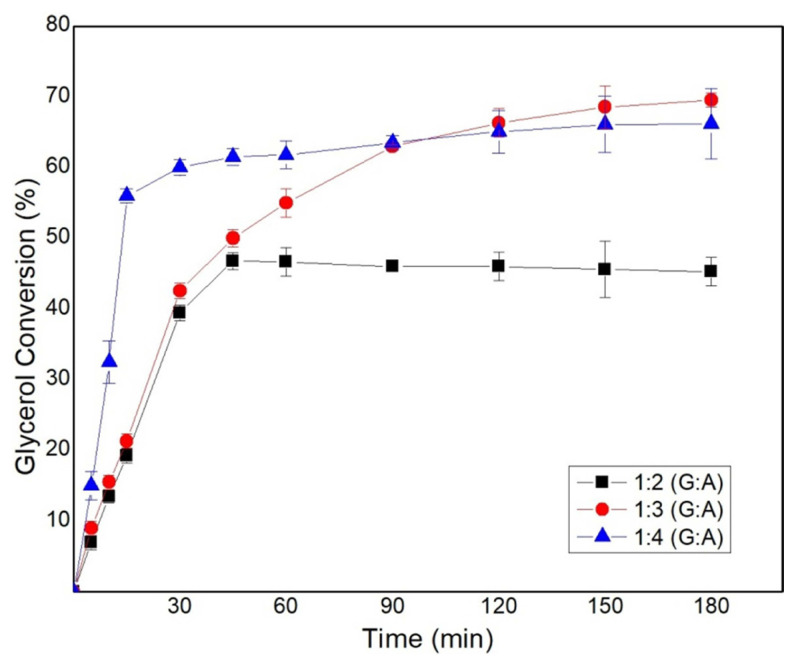
Effect of molar ratio (G:A) on the glycerol conversion,1 wt % catalyst loading, 323K, and 700 rpm.

The molar ratio of the reactants influences the rate of the reaction and its equilibrium conversion. This was investigated by varying the molar ratio of acetone from 1:2, 1:3, and 1:4 (G:A). Reactions were performed at 323 K and 700 rpm in order to lower the viscosity of glycerol and promote the mixing of the reactants. A catalyst loading of 1.0 wt% of the total weight of the reaction mixture was kept constant for each reaction and the results are as reported in Figure 1. It was observed that the increase in the molar ratio of acetone leads to a higher reaction rate as it aids in the swelling of the catalyst and also drives the forward reaction. It was observed that G:A–1:4 has the highest reaction rate in the first few minutes and approaches a conversion of 65% in 20 min without significant change further. The higher concentration of acetone aids in the miscibility of glycerol also makes the overall solution less viscous, hence reducing the overall diffusion limitations. The mole ratio of G:A–1:3 gave a conversion of 69% after a reaction time of 180 min. The slightly lower conversion at G:A–1:4 compared to 1:3 could be due to the dilution effect caused by the higher concentration of acetone, water, and solketal leading to lesser glycerol molecules to reach the active site and get adsorbed near acetone molecules for the forward reaction to happen according to the LHHW model. A similar drop in the conversion is also observed by Churipard et al. when the G:A molar ratio was increased from 1:5 to 1:6 [22]. The rate of reaction was found to be higher at higher concentrations of acetone as it not only promotes the forward reaction it also increases the homogeneity of the reaction mixture. An increase in the conversion after a certain threshold of reaction, could be attributed to the formation of water, making the biphasic liquid homogenous and due to the higher polarity of water, which aids in the swelling up of the gel-resin. Water has a higher affinity for forming protonated water at the sulphonic sites and further attracting polar compounds towards it. In solvent less condition the conversion of glycerol was low initially due to the low miscibility of glycerol and acetone later the reaction proceeded normal [24]. The molar ratio of 1:3 was found to be the optimal ratio to promote the forward reaction and further studies were directed with this molar ratio.

### 3.2. Effect of catalyst loading on solketal synthesis

**Figure 2:**
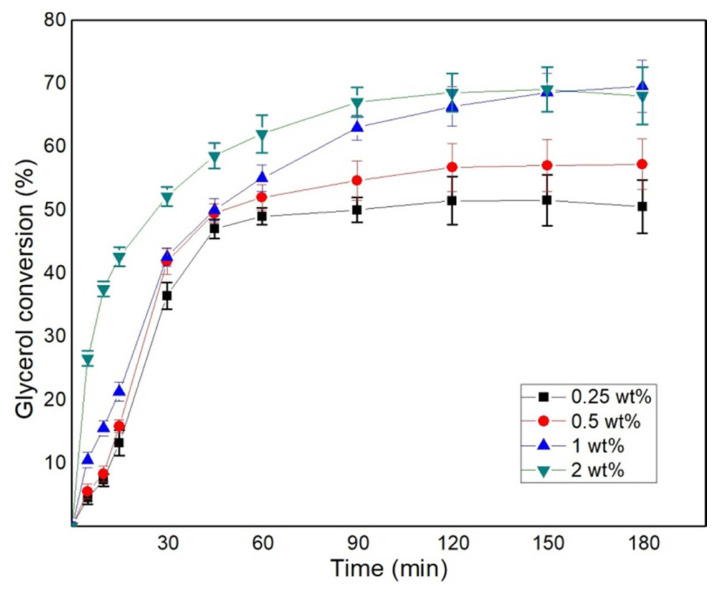
Effect of catalyst loading on glycerol conversion, G:A–1:3, 323K, and 700 rpm.

The influence of catalyst loading on glycerol conversion was studied by varying the weight of the catalyst from 0.25, 0.5, 1.0 to 2.0 wt% of the total reactant weight for the reactant ratio G:A–1:3 at 323 K and 700 rpm. Figure 2 indicates a significant effect on the overall conversion as the amount of the catalyst is varied. At 2.0 wt% catalyst loading, a high initial reaction rate was observed due to the presence of a greater number of acid sites. The simultaneous production of water, aids in the reactant miscibility and the swelling of the catalyst. However, the presence of higher acid sites also promotes the reverse reaction which can be observed by a drop in the glycerol conversion rate after 90 min. The presence of the higher overall concentration of the acidic sites in 2.0 wt% catalyst loading compared to 1.0 wt%, resulted in hydrolysis of solketal to take place and thus resulted in a slightly lower conversion. The pronounced effect of the reverse reaction was studied by Churipard et al. in an independent study where the reaction mixture composition after maximum conversion was precalculated and this mixture of reactants and products was subjected to ketalization reaction [22]. That increased the observed concentration of glycerol concentration at the end of the reaction which was due to solketal hydrolyses into glycerol in presence of water and acid catalyst. The addition of 1.0 wt% catalyst resulted in the highest conversion as it provides the ideal acid sites to promote the forward reaction and prevents the choking of the acid sites by water. The addition of 0.25 and 0.50 wt% catalyst results in a very slow reaction rate due to the lack of sufficient acid sites for the reaction to take place. As the reaction proceeds, the water produced deactivates some of the active sites and there is a marked drop in conversion. Water is known to have a high affinity towards the acidic sulphonic groups of an ion exchange resin and it blocks these sites from participating in the reaction. Churipard et al. observed a successive drop in glycerol conversion from 86% to 79% as the catalyst loading was increased from 0.5 wt% to 2.0 wt% of glycerol weight, due to the hydrolysis of solketal [22]. Sulistyo et al. showed for the glycerol ketalization with Amberlyst-15, that catalyst loading had minimal effect on conversion but as the loading increased beyond 5.0 wt% of glycerol, a drop in the conversion was observed [25].

### 3.3. Effect of temperature on solketal synthesis

**Figure 3:**
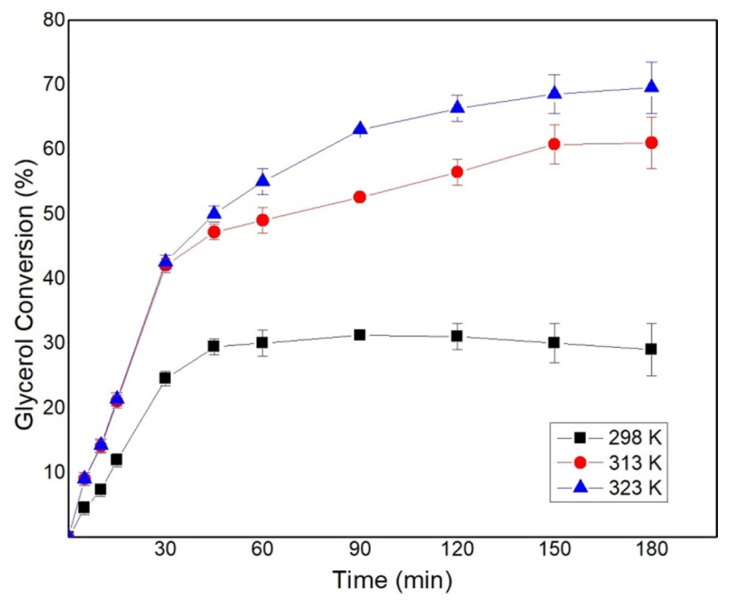
Effect of temperature on glycerol conversion conducted at G:A–1:3, 1wt% catalyst loading, and 700rpm.

The effect of temperature on the ketalization of glycerol was studied for a temperature range of 298–323 K, molar ratio G:A–1:3 and 1.0 wt% catalyst loading at 700 rpm. The variation of temperature has a significant impact on the conversion of glycerol (Figure 3). The conversion of glycerol at 298 K was the least due to the high viscosity of glycerol. A conversion of around 30% is observed at 298 K, which might be due to the partial miscibility of glycerol with acetone and the adsorption of glycerol onto the acid sites due to its polar nature. With acceleration in temperature, the conversion of glycerol increased to 67% for a reaction temperature of 323 K. Similar results were reported by Esteban et al. in a solvent-free condition [24]. Khayoon and Hameed reported an increase in glycerol conversion with an increase in the temperature while carrying out the reaction in a solventless medium and using Ni/Zr on activated carbon as the catalyst [26]. A similar trend was also observed by Sulistyo et al. who used Amberlyst-15 in the absence of a solvent for the ketalization reaction [25]. As the temperature increases the conversion increases and yields similar results to that of a solvent condition. The increase in conversion with the increase in the temperature suggests that the reaction is endothermic in nature.

The yield obtained by varying the temperature, mole ratio, and catalyst loading are provided in (Table 2). The yield varies significantly with the reaction conditions, the selectivity of solketal over the six-membered by-product is around 97% for all the reaction conditions. The yield obtained by Indion 225H are slightly higher than those obtained by Esteban et al., this is due to the presence of gel type resin which offers more pore volume than the macro porous resins [24]. Similarly, Faria et al. reported Amberlyst-15 has lower acidity than Amberlyst-35/47, it gives higher conversion due to its larger surface area and smaller particle diameter which aid in the internal diffusion [27].

### 3.4. Kinetic study

The reaction kinetics and thermodynamics provide more insight into the glycerol ketalization technology and are also vital in reactor designing for scaling up at an industrial scale. Kinetic studies for the ketalization reaction were carried out at G:A–1:3 molar ratio, 1.0 wt% catalytic loading, and variable temperature from 298–323 K. Equilibrium data was collected by allowing the reaction to occur for a long period of time and the equilibrium constant.


(4) $$K_{C}=\frac{C_{s}C_{w}}{C_{a}C_{g}}$$

where C_s_, C_w_, C_g,_ and C_a_ are the molar concentrations of solketal, water, glycerol, and acetone respectively.

**Figure 4:**
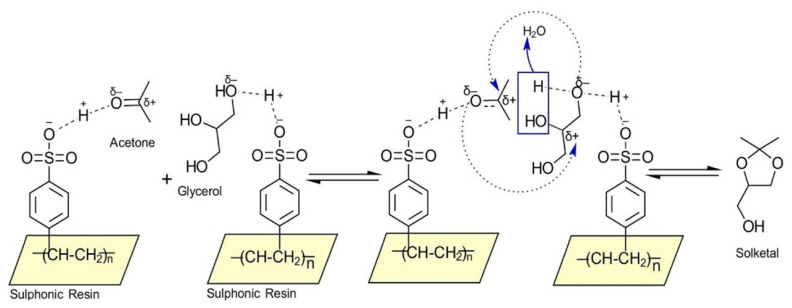
Reaction mechanism for the conversion of glycerol to solketal.

The reaction mechanism involves 1) adsorption of reactants on adjacent acid sites of the resin where a partial charge is imparted on the carbonyl group of acetone and the primary alcoholic group of glycerol. 2) The activated carbonyl group attacks the alcoholic group and simultaneous dehydration of the secondary alcoholic group of glycerol occurs. This electron-deficient carbon undergoes cyclization to form the five-membered ring having protected oxygen. 3) Desorption of the products from the active sites (Figure 4). The Langmuir-Hinshelwood-Hougen-Watson (LHHW) model was used to describe the theoretical nature of reaction as it is highly reliable and accurate model used to describe a catalytic reaction. The developed model is usually consistent with the experimental data within a narrow error range. The LHHW model assumes that the adsorption of both acetone and glycerol onto adjacent active sites and a bimolecular reaction takes place. The formation of the intermediate compounds and reactant diffusion is assumed to occur without any resistance while the formation of solketal is considered as the rate controlling step. The reaction steps and their respective rate equations involved are described below:

1. Adsorption of reactants onto the active site (G: glycerol, A: acetone, X: active site):
  Rate of adsorption of reactants is given by Eq. (5) and Eq. (6).
(5) $$G+X\overset{k_{g}}{\underset{k_{-g}}{⇌}}GX;\;\;\;r_{ga}=K_{g}(C_{g}\bar{C_{m}}-\frac{\bar{C_{g}}}{k_{g}})$$
(6) $$A+X\overset{k_{a}}{\underset{k_{-a}}{⇌}}AX;\;\;\;r_{aa}=K_{a}(C_{a}\bar{C_{m}}-\frac{\bar{C_{a}}}{k_{a}})$$
2. Surface reaction on the dual sites and the formation of products (S: solketal, W: water):
(7) $$GX+AX\overset{k_{s}}{\underset{k_{-s}}{⇌}}SX+WX;\;\;\;r_{s}=K_{s}(\bar{C_{g}C_{a}}-\frac{\bar{C_{s}C_{w}}}{k_{s}})$$
3. Desorption of products:
  The rate of desorption of products is given by Eq. (8) and Eq. (9).
(8) $$SX\overset{k_{sd}}{\underset{k_{-sd}}{⇌}}S+X;\;\;\;r_{sd}=K_{sd}(\bar{C_{s}}-\frac{\bar{CmCs}}{k_{g}})$$
(9) $$WX\overset{k_{wd}}{\underset{k_{-wd}}{⇌}}W+X;r_{wd}=K_{wd}(\bar{C_{w}}-\frac{\bar{CmCw}}{k_{g}})$$
(10) $${\text{C}}_{\text{Total}}=\bar{{\text{C}}_{\text{m}}}+{\text{K}}_{\text{a}}{\text{C}}_{\text{a}}\bar{{\text{C}}_{\text{m}}}+{\text{K}}_{\text{g}}{\text{C}}_{\text{g}}\bar{{\text{C}}_{\text{m}}}+{\text{K}}_{\text{s}}{\text{C}}_{\text{s}}\bar{{\text{C}}_{\text{m}}}+{\text{K}}_{\text{w}}{\text{C}}_{\text{w}}\bar{{\text{C}}_{\text{m}}}$$

The bar above the concentration term indicates the concentration of adsorbed species. $\bar{C_{m}}$ is the concentration of the active sites available while C_Total_ is the total active sites at any time and represented by the Eq. (10) and Eq. (11). Assuming adsorption and desorption rates r_aa_, r_ga_, r_sd,_ and r_wd_ to be zero, the equilibrium constant from Eq. (4), and rearranging the surface reaction rate Eq. (7), following LHHW model is obtained (Eq. (12)):


(11) $$\bar{C_{m}}=\frac{C_{Total}}{1+K_{a}C_{a}\bar{C_{m}}+K_{g}C_{g}\bar{C_{m}}+K_{s}C_{s}\bar{C_{m}}+K_{w}C_{w}\bar{C_{m}}}$$

(12) R=K(CaCg-CsCwKc){1+KaCa+KgCg+KsCs+KwCw}2

Previous studies have been conducted by neglecting the adsorption constants of acetone and glycerol because of the fact that the adsorption of water is significantly higher. Considering the low polarity of solketal, which lowers its affinity towards the acid sites, the final rate equation proposed Eq. (13):


(13) R=K(CaCg-CsCwKc){1+KwCw}2

The net rate of disappearance of glycerol is defined by the equation:


(14) $$R=\frac{dX_{G}}{dt}\frac{n_{G}}{W_{cat}}$$

This kinetic model represented by the ode Eq. (14) was initially solved using the ode45 solver in MATLAB. The ode solver was nested with the GA function and the appropriate constraints were provided to search in the positive integers. A population size of 100 was chosen as the number of unknown variables was 4, while the elite count was set to 0.5× population size and a crossover fraction of 0.8 was set to generate the next population. The objective function to be optimized calculates the residual sum of squares and is defined by Eq. (15).


(15) $$\text{f}=\sum_{i=0}^{n}\left(C_{g,Exp}^{i}-C_{g,Thr}^{i}\right)$$

where n is the number of data points collected, C_g,Exp_ and C_g,Thr_ are the experimental and theoretical concentrations of glycerol for the i^th^ run.

**Figure 5:**
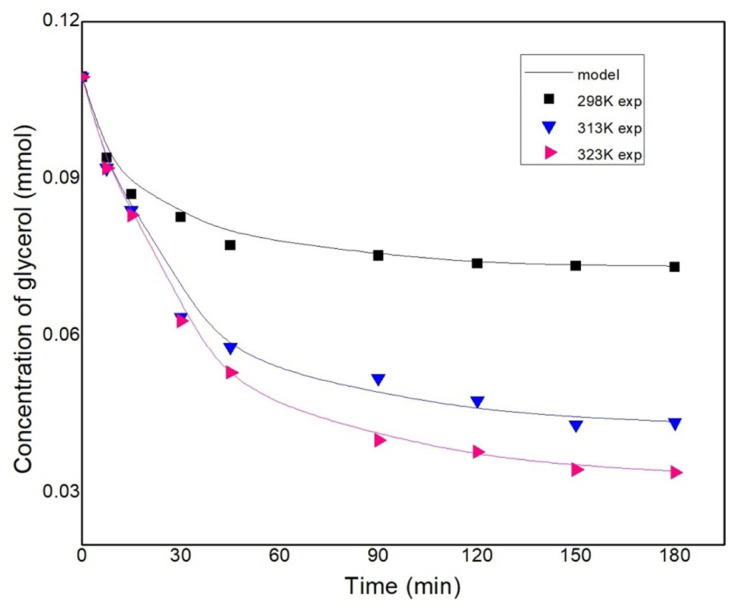
Kinetic study conducted at G:A–1:3, 1 wt% loading and fitted against the LHHW.

The model showed a good fit with the experimentally determined results and the estimated kinetic constants at all three temperatures are provided in Figure 5. The obtained values are reported in Table 3. The rate constant k was found to have slightly lower values compared to those obtained by Nanda et al. while using the macroporous Amberlyst-35 in a solvent medium. The use of a gel-type resin and a solventless medium could offer initial limitations in swelling and homogeneity of the reaction medium leading to a slower reaction rate, while the adsorption constant for water was found to be higher as the gel-resins show a higher affinity towards water.

The adsorption constant for water is significantly higher than the adsorption constants for acetone and glycerol, especially at lower temperatures. At lower temperatures (298 K) the adsorption of acetone and glycerol is overshadowed by the higher affinity of water which is observed by the drop in conversion at 60 min (Figure 4), while at higher temperatures (313 K–323 K), the adsorption constants of acetone and glycerol are higher and are noticed by the higher reaction rate compared to lower temperatures. Furthermore, the adsorption constant of acetone is higher than glycerol.

**Figure 6:**
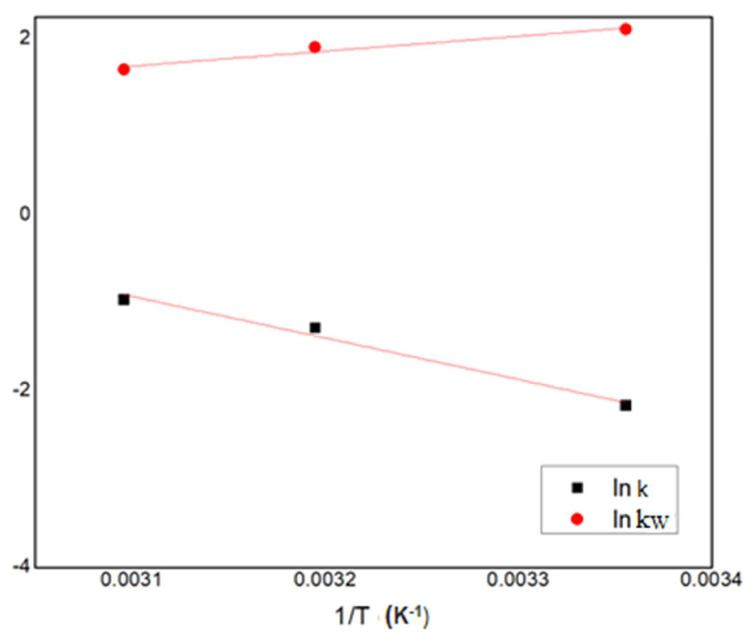
Kinetic parameters of the LHHW model plotted against 1/T (K^−1^).

The estimated rate constants were used to determine the activation energy using Arrhenius equation while the adsorption constant for water was used to determine the enthalpy of adsorption using equations Eq. (16), Eq. (17), and the log plots in Figure 6.


(16) $$\text{k}={\text{k}}_{\text{o}}\;\text{exp }(-\frac{E_{a}}{RT})$$

(17) $${\text{k}}_{\text{w}}={\text{k}}_{\text{h}}\;\text{exp}(\frac{\Delta H_{w}}{RT})$$

The activation energy E_a_ was found to be 39.36 kJ/mol and the ΔH_w_ = −23.1786 kJ/mol and are in a similar range to those reported in the literature. Nanda et al. reported activation energy of 55.3 kJ/mol [19], while Rossa et al. reported activation energy for forward reaction to be 44 kJ/mol for the catalyst BEA zeolite [28] and Moreira et al. reported activation energy of 67.4 kJ/mol while considering a diffusion-based model [29]. The enthalpy of adsorption of water was also in the similar range obtained by Nanda et al. (ΔH_w_ = −30 kJ/mol) but their kinetic constant for adsorption of water was much lower [19].

## 4. Conclusion

The ketalization reaction between glycerol and acetone to produce solketal, was carried out in the presence of an acidic cation ion exchange resin Indion 225H in solventless condition and a 70% conversion of glycerol was achieved. These results were optimized at a G:A–1:3 molar ratio, 323 K, and 1.0 wt% catalyst loading. The establishment of the reaction mechanism according to LHHW, showed that reaction is the rate controlling step and the activation energy of the reaction was found to be 39.3 kJ/mol. The kinetic model available can be successfully used to simulate the ketalization process for the continuous production of solketal. The overall work suggests the use of Indion 225H as the catalyst for the continuous production of solketal which is economically beneficial and can be commercially ventured.

## Supplementary Material

Figure S1 Solketal yield obtained at various reaction conditions.

Figure S2 (A) MATLAB source code for solving kinetics using Genetic Algorithm.

## Figures and Tables

**Table 1:** Characteristics of Indion 225H.

• Appearance	• Golden yellow beads
• Matrix	• Styrene divinylbenzene copolymer
• Functional group	• Sulphonic acid (SO_3_^−^)
• Ionic form	• H^+^
• Total ion exchange capacity	• 1.8 meq/mL, minimum
• Moisture holding capacity	• 49%–55%
• Particle size	• 0.3–1.2 mm
• Shipping weight	• 780 kg/m^3^

**Table 2:** Yield of Solketal at various reaction conditions.

Reaction conditions (G:A, catalyst loading, temperature)	Yield of Solketal
1:3, 1 wt%,323 K	68%
1:3, 2 wt%, 323 K	38%
1:3, 1.25 wt%,323 K	41%
1:3, 1 wt%,298 K	30%
1:3, 1 wt%,313 K	50%
1:4, 1 wt%,323 K	60%

**Table 3:** Kinetic parameters obtained after fitting the theoretical model.

Temp (K)	K (L^2^mol^−^ g_cat_^−^ min^−^)	K_W_	R^2^
298	0.115	8.2	0.987
313	0.278	6.71	0.978
323	0.383	5.214	0.975
