# Supplementary figures and images for: Parametric and kinetic study of solvent-free synthesis of solketal using ion exchange resin
Source: Turk J Chem. 2022 Feb 23;46(3):881–9. doi: 10.55730/1300-0527.3376 (PMC10503983; doi:10.55730/1300-0527.3376)

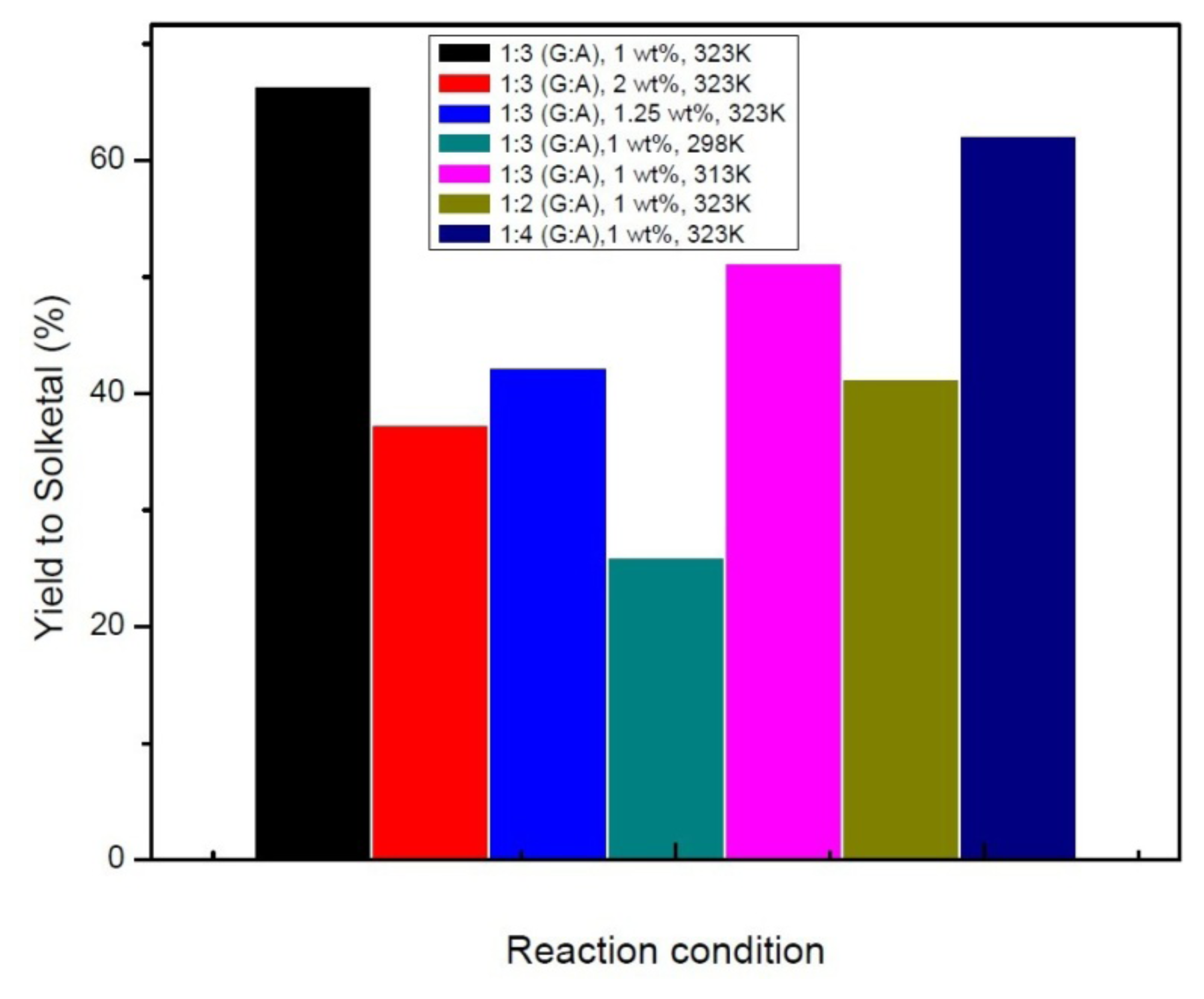

Supplement: Figure S1 — Solketal yield obtained at various reaction conditions. [file turkjchem-46-3-881s1.tif]

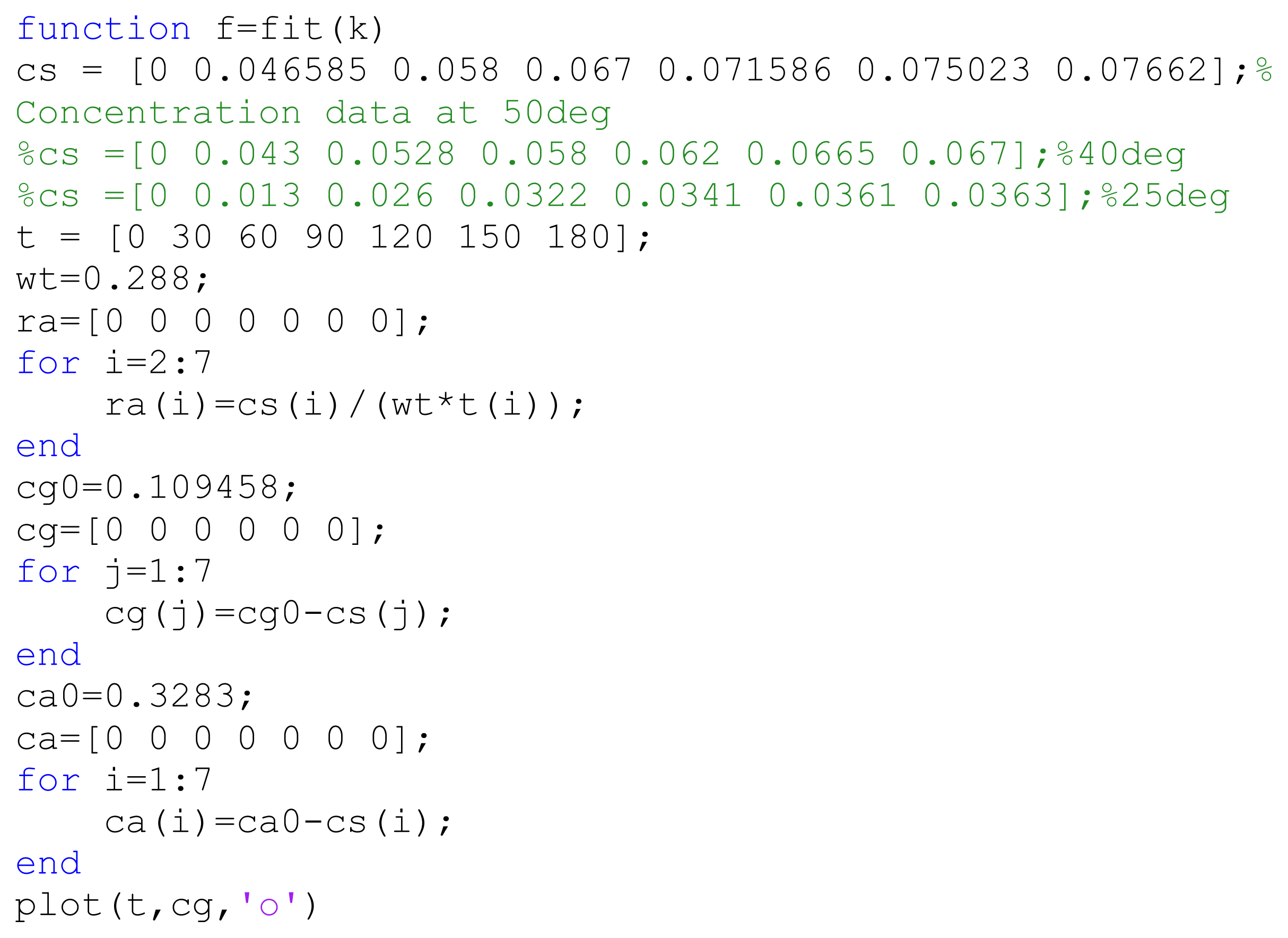

Supplement: Figure S2 — (A) MATLAB source code for solving kinetics using Genetic Algorithm. [file turkjchem-46-3-881s2a.tif]

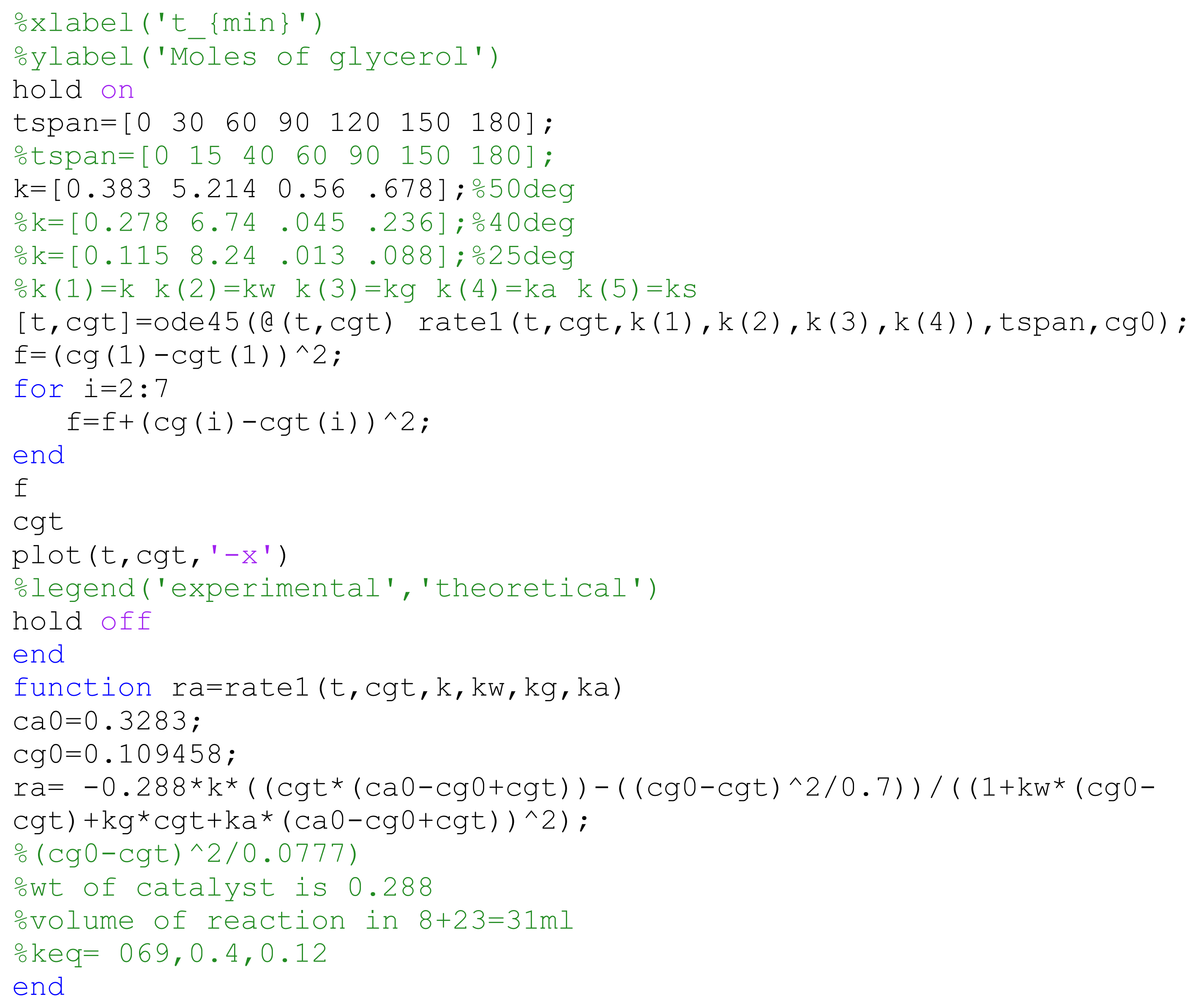

Supplement: Figure S2 — (A) MATLAB source code for solving kinetics using Genetic Algorithm. [file turkjchem-46-3-881s2b.tif]
